# Antiseptic Treatment and Disinfection of Scarlet Fever: Remarks on the Method of Using Inunction

**Published:** 1893-08-05

**Authors:** J. Brendon Curgenven


### ANTISEPTIC TREATMENT AND DISINFECTION OF SCARLET FEVER; REMARKS ON THE METHOD OF USING INUNCTION, &c.

By J. Brendon Curgenven, M.R.C.S.

It is apparent from the observations published on this subject in the medical journals, and from letters I have received, that not a few medical men have failed to understand the method of carrying out the disinfection in all its details, partly perhaps because they have not read my papers on the subject, and partly to my having illustrated some of the more important points by cases only.

The inunction must not be regarded as a sort of conjuring trick, that when performed the infection disappears, as the watch from the hat; it is only one part of the treatment which in all its details must be carried out with every care.

The antiseptic used, the "oleusaban" eucalyptus disinfectant "A," is entirely and rapidly volatile, consequently it does not remain long on the skin, nor does it obstruct or interfere with its proper action. It contains no fixed oil, water, nor spirit.

Every portion of the skin must be lightly smeared over with the antiseptic, using the hand and fingers for the purpose. No portion of cuticle must be omitted; under the nails, between the toes, the intricacies of the ears, and other spots must receive attention. The cuticle is thus thoroughly disinfected, for the essential oil is very penetrating, and much is absorbed and carried into the circulation. If inunction is commenced on the first or second day of the fever, the disease is in many cases aborted, and in others the symptoms are greatly mitigated. The chance of others becoming infected is also lessened. If the treatment can be commenced during the initial stage of headache, sore throat, and sickness, the fever will in most cases be arrested.

Inunction should be used night and morning for three days, and then each night for a week, after a warm bath. In mild cases it is not necessary to continue it longer, but in severe cases, and where there is much desquamation it may be continued for another week.

Of equal importance to inunction is the use of the antiseptic to disinfect the breath and the mucous membrane of the air passages. The air around the patient must be kept saturated with the vapour night and day without intermission for at least ten days. Air, like water, will only dissolve a certain definite amount of the oil. One litre of air dissolves 0·0286 of a gramme of the oil of eucalyptus globulus, and this state of saturation must be maintained to destroy all infection in the breath. The pillow and sheets should be sprinkled and a spray diffuser used several times during the day and night. A handkerchief moistened with the disinfectant may also be on the bed near the patient, and if the room be large the bed should be surrounded by a canopy and screen of sheets. Should the patient complain of headache, diminish the amount used and admit more fresh air.

The mucous membrane of the throat, stomach, and intestines receives a large amount of the poison. The throat can be sprayed or brushed with the disinfectant, but this is unnecessary in most cases as the inhaled vapour and the medication is sufficient. But to reach the stomach and intestines the disinfectants or the globulus oil should be given in from two to ten drop doses, according to age, in mucilage sweetened with syrup three times a day. The evacuations and urine should be passed into a vessel in which a teaspoonful of the disinfectant has been placed, covered with a towel, and removed.

Any susceptible persons or children who have been exposed to infection before inunction was commenced, should be confined to a room, the air of which is kept saturated with the vapour of the disinfectant for three days, and their clothes and bedlinen sprinkled with it. They should also take the emulsion. If there is not a second room they can remain in the room with the patient.

The clothes worn by the patient before and when taken ill, his toys, books, &c., must be disinfected by being well sprinkled with the "oleusaban," and closely shut up in a box or cupboard for three days, repeating the sprinkling each day. His bed and body linen can be treated in the same way, but that used by him while under treatment, and his bed, are disinfected by the process.

The degree of saturation by the vapour, given off at the ordinary temperature, is of sufficient strength to destroy the bacilli of anthrax in 72 hours, whilst air saturated with the vapour of carbolic acid at the ordinary temperature has no effect on the bacilli of any disease; nor will a 10 per cent. solution of the latter in olive oil destroy bacilli.

The "B" oleusaban, being an emulsion, mixes readily with water. I have found this useful to put into the water used for washing the floors, furniture, utensils, linen, &c. A tablespoonful can be put into the chamber utensils before they are used instead of the "A," and it can be used in the closet. It can be also put into the water for washing hands, and into the bath; it can be used as a gargle, and as a wash for the hair.

Inflamed glands and rheumatic joints should be rubbed with the "A" disinfectant. Should there be any rhinorrhœa or otorrhœa spray the passages; and abscesses of the glands are best cleansed and dressed with the "oleusaban" emulsion.

If fresh cases arise in a family or school that cannot be ascribed to infection from those under treatment before inunction was commenced, some other source of infection must be sought for. A mild case, with only a sore throat, may have escaped notice. All should be examined by the aid of a lens for desquamation on the hands, feet, and back; I have found cases desquamating attending school, serving behind the counter, and in other ways following their occupations, and the fact of their having scarlet fever was unknown to them or their friends. New-made articles of clothing may be the source, as most of the tailoring and dressmaking is done at the houses of the workers; and it must be in the experience of many to have found cases of scarlet fever in the work-rooms. I found a dressmaker once making two ball dresses and at the same time nursing the child who was covered with the rash of scarlet fever. The milk and food supply should receive attention, and any other external source of infection that may be thought of.

The vomit at the commencement of the attack, and the clothes, carpet, floor, or utensil soiled by it, must be disinfected; in no case scarcely is this attended to.

During outbreaks in large schools the dormitories should be sprayed and the pillows and sheets sprinkled with the disinfectant every night. It would be well to do this also in families of children.

In the treatment and disinfection of diphtheria it is most important to keep the air around the patient saturated with the vapour of the disinfectant. He should have it rubbed over the chest and the front of the neck every four hours, that the warmth of the skin may volatilise it in a favourable position for inhalation. The bed must be sprinkled and the spray diffuser used frequently. The steam spray diffuser or the bronchitis kettle are useful aids in diffusing the vapour. If the room be large the bed should be surrounded by a canopy and screen of sheets. The emulsion should be given and the throat sprayed or brushed with the "oleusaban." The source of the infection of diphtheria must be sought for in the dusthole, kitchen-middens, drains, or water supply; also in uncleaned cow, fowl, or pigeon houses. Small-pox, measles, and chicken-pox should be treated as scarlet fever, but it will be sufficient for measles to be inuncted once a day. In mumps and whooping cough it is more difficult to prevent infection, because the patients are not, as a rule, confined to their beds. Besides having the fronts of their clothes sprinkled and the room sprayed, they should wear a small muslin bag of cotton or wood wool suspended from the neck on the chest, saturated twice a day with the "oleusaban." In mumps the swollen glands must be rubbed with it two or three times a day, and in both cases their pillows and sheets should be sprinkled at night and the spray used at intervals. They should also take the emulsion in whooping cough, a dose being given at bedtime, another in the night, and one in the day. Other sedative medicines can be given in severe cases.

It must not be considered that this method of antisepsis is perfect, and that there will not be some apparent failures. The treatment I consider is yet in its infancy, and it requires the experience and observations of many to perfect its application to all infectious diseases. It must also be borne in mind that we are combating an invisible enemy, and that there exist many loopholes for its escape. Nevertheless, the results obtained are far better than those shown by the isolation hospital system in checking the spread of infection, in mitigating the severity of the disease, in lessening the frequency of complications, and in lowering the mortality. For these points I refer your readers to my last paper published in the *Medical Magazine*, and separately by Messrs. Southwood, Smith, and Co.

